# Fixel-Based Analysis Reveals Tau-Related White Matter Changes in Early Stages of Alzheimer's Disease

**DOI:** 10.1523/JNEUROSCI.0538-23.2024

**Published:** 2024-04-02

**Authors:** Khazar Ahmadi, Joana B. Pereira, Danielle van Westen, Ofer Pasternak, Fan Zhang, Markus Nilsson, Erik Stomrud, Nicola Spotorno, Oskar Hansson

**Affiliations:** ^1^Clinical Memory Research Unit, Department of Clinical Sciences, Lund University, Lund 22362, Sweden; ^2^Institute of Cognitive Neuroscience, Faculty of Psychology, Ruhr University Bochum, Bochum 44801, Germany; ^3^Division of Neuro, Department of Clinical Neurosciences, Karolinska Institutet, Stockholm 17176, Sweden; ^4^Diagnostic Radiology, Department of Clinical Sciences, Lund University, Lund 22185, Sweden; ^5^Departments of Psychiatry, Brigham and Women’s Hospital, Harvard Medical School, Boston, Massachusetts 02115; ^6^Radiology, Brigham and Women’s Hospital, Harvard Medical School, Boston, Massachusetts 02115; ^7^Department of Psychiatry, Massachusetts General Hospital, Harvard Medical School, Boston, Massachusetts 02114; ^8^Department of Medical Radiation Physics, Lund University, Lund 22185, Sweden; ^9^Memory Clinic, Skåne University Hospital, Malmö 21428, Sweden

**Keywords:** Alzheimer's disease, diffusion tensor imaging, fixel-based analysis, memory, white matter

## Abstract

Several studies have shown white matter (WM) abnormalities in Alzheimer's disease (AD) using diffusion tensor imaging (DTI). Nonetheless, robust characterization of WM changes has been challenging due to the methodological limitations of DTI. We applied fixel-based analyses (FBA) to examine microscopic differences in fiber density (FD) and macroscopic changes in fiber cross-section (FC) in early stages of AD (*N* = 393, 212 females). FBA was also compared with DTI, free-water corrected (FW)-DTI and diffusion kurtosis imaging (DKI). We further investigated the correlation of FBA and tensor-derived metrics with AD pathology and cognition. FBA metrics were decreased in the entire cingulum bundle, uncinate fasciculus and anterior thalamic radiations in Aβ-positive patients with mild cognitive impairment compared to control groups. Metrics derived from DKI, and FW-DTI showed similar alterations whereas WM degeneration detected by DTI was more widespread. Tau-PET uptake in medial temporal regions was only correlated with reduced FC mainly in the parahippocampal cingulum in Aβ-positive individuals. This tau-related WM alteration was also associated with impaired memory. Despite the spatially extensive between-group differences in DTI-metrics, the link between WM and tau aggregation was only revealed using FBA metrics implying high sensitivity but low specificity of DTI-based measures in identifying subtle tau-related WM degeneration. No relationship was found between amyloid load and any diffusion-MRI measures. Our results indicate that early tau-related WM alterations in AD are due to macrostructural changes specifically captured by FBA metrics. Thus, future studies assessing the effects of AD pathology in WM tracts should consider using FBA metrics.

## Significance Statement

Diffusion tensor imaging (DTI) has been widely used to study white matter (WM) integrity in Alzheimer's disease (AD). However, the methodological shortcomings of DTI limit an accurate biological interpretation. We used fixel-based analysis (FBA) to assess fiber-specific WM degeneration and its correlation with the underlying pathology and cognitive symptoms in early AD. Our results revealed that elevated tau- but not Aβ-PET uptake in the medial temporal structures was correlated with atrophy of the parahippocampal portion of the cingulum bundle. The tau-related damage in this WM bundle was further linked to memory deficits. Importantly, the tau-WM correlation was not detected by tensor-derived measures. These findings suggest that FBA metrics may serve as a biomarker for early detection of tau pathology in AD.

## Introduction

Alzheimer's disease (AD) is characterized by the accumulation of amyloid-beta (Aβ) plaques and neurofibrillary tangles of hyperphosphorylated tau (Jack et al., 2013; Hansson, 2021). Although AD is typically considered a gray matter (GM) disease (Bejanin et al., 2017; Iaccarino et al., 2018), increasing evidence shows concomitant white matter (WM) abnormalities (Englund, 1998; Amlien and Fjell, 2014; Lo Buono et al., 2020). Diffusion-weighted MRI (dMRI) has enabled noninvasive investigation of WM integrity in AD, with most studies relying on diffusion tensor imaging (DTI; Amlien and Fjell, 2014)]. However, the literature describing the link between WM changes and AD pathology is conflicting. While the results of some DTI studies suggest an association between lower mean diffusivity (MD) and higher fractional anisotropy (FA) with Aβ pathology in early AD (Racine et al., 2014), others have shown an opposite correlation (Chao et al., 2013). Conversely, there are reports of no association between Aβ and WM alterations (Kantarci et al., 2014; Strain et al., 2018). These inconsistent findings may reflect cohort-specific differences, but they might also arise from the methodological challenges of DTI that in turn may affect its biological specificity. Due to partial volume effects, the DTI metrics are not specific to a single tissue type. This is particularly relevant in AD where vasogenic edema results in an increase in extracellular free water (FW) in the brain. This flaw can be mitigated using FW-DTI providing more accurate tissue-based metrics (Pasternak et al., 2009; Bergamino et al., 2021). Further, diffusion in DTI is modeled based on the assumption that the displacement of water molecules has a Gaussian distribution while this assumption may not hold in biological tissues (Jensen and Helpern, 2010). The contribution of non-Gaussian diffusion can be quantified using diffusion kurtosis imaging (DKI), which is shown to be potentially more sensitive than DTI in detecting microstructural WM changes (Gong et al., 2013; Zhang et al., 2021).

Another limitation of DTI is the inability to resolve multiple fiber orientations in regions with crossing fibers (Jeurissen et al., 2013). The fixel-based analysis (FBA) framework facilitates fiber-tract–specific statistical comparisons (Raffelt et al., 2015, 2017) using a higher-order diffusion model known as constrained spherical deconvolution (Tournier et al., 2007, 2008) that enables the characterization of multiple fiber orientations in a voxel. FBA provides metrics estimated from fixels, that is, distinct fiber populations within a voxel. These quantitative metrics include fiber density (FD) reflecting microscopic changes in intra-axonal volume, fiber cross-section (FC), an index of macroscopic alterations in a cross-sectional area perpendicular to WM bundles, and a combined measure, FDC, as the product of FD and FC. In the first application of FBA in patients with mild cognitive impairment (MCI) and AD, Mito et al. (2018) found fiber-specific alterations in tracts connecting key regions affected by AD. However, they did not observe an association between FBA metrics and Aβ accumulation. Furthermore, in their study, the association between WM and tau pathology was not investigated. The available studies on such associations are mainly based on DTI and conducted on relatively small cohorts (Strain et al., 2018; Carlson et al., 2021; Pereira et al., 2021). Hitherto, two studies have assessed the association of FBA metrics with tau pathology reporting mixed results. Specifically, one study has shown an inverse relationship between FC in the ventral cingulum and increased tau-PET uptake in the entorhinal cortex (EC; Luo et al., 2021) whereas in the other study, tau-PET uptake was not associated with FBA metrics when accounting for Aβ deposition (Dewenter et al., 2023). Given those inconsistencies, we aimed to examine the extent of WM degeneration in early AD and its correlation with the underlying pathology and cognitive performance. This is particularly timely, given that the emerging disease-modifying therapies targeting Aβ and tau will likely be more effective during the early stages of AD (Hansson, 2021) while effective markers of subtle neurodegeneration are still lacking. In this study, we focused on a large cohort of nondemented individuals and applied FBA, DTI, FW-DTI, and DKI to investigate potential early signs of WM degeneration.

## Materials and Methods

### Participants

This study comprised cognitively unimpaired individuals (CU) and MCI patients, recruited from the Swedish BioFINDER-2 study (NCT03174938) that has been previously described in detail (Leuzy et al., 2020; Palmqvist et al., 2020). The diagnosis of MCI was made by physicians specialized in cognitive disorders, based on the participants’ neuropsychological performance (Palmqvist et al., 2020). They were further stratified into Aβ-negative or Aβ-positive groups based on the CSF Aβ42/40 ratio (Pichet Binette et al., 2022). Note that Aβ-negative MCI patients were not included in the present study. Of 484 initially included participants, a total of 91 individuals were excluded due to excessive motion or other types of imaging artifacts (*N* = 13), evidence of severe vascular copathology such as cerebral infarcts (*N* = 10), and extensive white matter hyperintensities (WMH; *N* = 68) that can affect the dMRI metrics (Svärd et al., 2017). Thereby, the final sample size consisted of 393 individuals. To visually rate the severity of the deep and periventricular WMHs, we used the Fazekas scale with scores ranging from 0 to 3 (Fazekas et al., 1987). The average Fazekas score in the excluded individuals was 2.73 (median and mode values, 3 and 3, respectively) indicating large confluent lesions. A summary of the participants’ demographic and clinical characteristics is provided in Table 1. All participants gave written informed consent. The study procedures were in accordance with the Declaration of Helsinki and were approved by the Ethical Review Board in Lund, Sweden.

**Table 1.** Participants’ characteristics

	Aβ-negative CU	Aβ-positive CU	Aβ-positive MCI	Statistic
	*N* = 224	*N* = 91	*N* = 78	
Age	65.41 (9.74)	69.97 (8.59)	71.97 (6.44)	*F* = 19.26
				*p* < 0.00001
Males/females	99/125	40/51	42/36	*χ*^2^ = 106.04
				*p* < 0.00001
APOE ε4 carriers%	35.26%	70.32%	75.64%	*χ*^2^ = 155.6
				*p* < 0.00001
Years of education	12.87 (3.59)	12.48 (3.32)	12.50 (4.45)	*F* = 0.507
				*p* = 0.603
ADAS-Cog delayed word recall	2.41 (1.71)	3.17 (1.96)	7.14 (2.20)	*F* = 184.7
				*p* < 0.00001
VOSP cube analysis	9.63 (0.85)	9.60 (1.12)	8.59 (2.27)	*F* = 18.89
				*p* < 0.00001
TMT (B–A) (seconds)	44.19 (27.06)	56.30 (52.14)	139.39 (109.87)	*F* = 74.23
				*p* < 0.00001
Global Aβ (SUVR)	0.47 (0.04)	0.65 (0.14)	0.78 (0.17)	*F* = 248.4
				*p* < 0.00001
Entorhinal tau (SUVR)	1.09 (0.12)	1.30 (0.26)	1.58 (0.37)	*F* = 133.1
				*p* < 0.00001

Data are presented as mean values followed by (standard deviations). Demographic and clinical variables were compared between groups using ANOVA or *χ*^2^ tests. CU, cognitively unimpaired; MCI, mild cognitive impairment; APOE ε4, apolipoprotein E ε4 allele; ADAS, Alzheimer's Disease Assessment Scale–Cognitive Subscale; VOSP, Visual Object and Space Perception Battery; TMT (B–A), trail making test Part B–A; SUVR, standardized uptake value ratio. Note that a higher ADAS score indicates poor memory performance.

### Neuropsychological assessment

Different cognitive domains were assessed in the present study. Memory performance was measured using the 10-word delayed recall test from the Alzheimer's Disease Assessment Scale–Cognitive Subscale (ADAS-Cog). Cube analysis from the Visual Object and Space Perception Battery (VOSP) was used to examine visuospatial abilities. Finally, the difference in the scores of trail making test (TMT) Parts B and A was selected as a measure of executive functions. Subtracting the time to complete Part A from Part B reduces visuospatial and working memory demands, hence providing a relatively pure indicator of executive/attention performance (Tideman et al., 2022).

### MRI acquisition and processing

All participants underwent diffusion-weighted magnetic resonance imaging (dMRI) using a 3T Magnetom Prisma scanner with a 64-channel receiver coil array, operating under syngo MR E11 software (Siemens Healthcare). The data were acquired using a single-shot EPI sequence with a multishell scheme using the following parameters: 2, 6, 32, and 64 gradient directions at *b* values of 0, 100, 1,000, and 2,500 s/mm^2^, respectively; isotropic resolution, 2 mm^3^; phase-encoding direction, A-P; FOV = 220 × 220 × 124 mm^3^; multiband factor = 2; parallel imaging factor = 2; TR = 3,500 ms; TE = 73 ms; and TA = 6:23 min. A second dMRI scan was also obtained with a reverse phase-encoding and seven gradient directions (1 × *b* = 0 and 6 × *b* = 1,000 s/mm^2^) for correction of susceptibility-induced distortions. Furthermore, a whole-brain T1-weighted scan (MPRAGE sequence, TR = 1,900 ms, TE = 2.54 ms, voxel size = 1 × 1 × 1 mm^3^, FOV = 256 × 256 × 176 mm^2^, TA = 5:15 min) and a T2-weighted FLAIR scan (TR = 5,000 ms, TE = 393 ms, TA = 4:37 min, same resolution and FOV as for the T1-weighted image) were acquired.

Preprocessing of dMRI data comprised denoising and Gibbs unringing implemented via DIPY tools “*dipy_denoise_patch2self*” and “*dipy_gibbs_ringing*” (Garyfallidis et al., 2014; Fadnavis et al., 2020), susceptibility off-resonance distortion, eddy current and head motion correction using FSL routines “*topup*” and “*eddy_cuda*” with outlier replacement (Andersson et al., 2003; Andersson and Sotiropoulos, 2016), and bias field correction via “*dwibiascorrect*” function in MRtrix3 (Tournier et al., 2019). Note that the head motion estimates were comparable across groups (Aβ-negative CU: mean = 0.4979 mm, SD = 0.2572 mm; Aβ-positive CU: mean = 0.4579 mm, SD = 0.2027 mm; and Aβ-positive MCI: mean = 0.5604 mm, SD = 0.2671 mm). Although all the above preprocessing steps can be performed with MRtrix3 tools, we chose to apply the denoising algorithm of DIPY as it does not make any prior assumptions about the signal structure and only relies on the randomness of the noise (Fadnavis et al., 2020).

dMRI data were then quality controlled at a subject level using the FSL tool “*eddy_quad*.” WMHs were automatically segmented in FLAIR images using the lesion prediction algorithm implemented in the lesion segmentation toolbox (https://www.applied-statistics.de/lst.html) of SPM. Moreover, gray matter (GM) and total intracranial volumes were estimated as part of T1-weighted image processing in FreeSurfer (v6; https://surfer.nmr.mgh.harvard.edu/).

### FBA processing

FBA was performed according to the recommended pipeline of MRtrix3 (Raffelt et al., 2017; Tournier et al., 2019). Briefly, tissue response functions for GM, WM, and CSF were computed using “dhollander” algorithm (Dhollander et al., 2016) based on which an average response function was obtained per tissue type across participants. Afterward, dMRI data were upsampled to an isotropic voxel size of 1.3 mm^3^. Fiber orientation distributions (FOD) were estimated via the “multishell multitissue”-constrained spherical deconvolution (MSMT-CSD) using the group-averaged tissue response functions (Jeurissen et al., 2014). Subsequently, a multitissue informed log-domain intensity normalization was applied to achieve comparable FOD amplitudes between participants. Next, in accordance with previous studies (Mito et al., 2018; Lyon et al., 2019; Zarkali et al., 2020; Savard et al., 2022) and based on MRtrix3 recommendations, an unbiased center-specific WM FOD template was generated from 30 randomly selected representative participants to which all individual FOD images were nonlinearly registered. The resulting transformed FODs were segmented to create discrete fixels. FD was calculated as the integral of the warped FOD lobe corresponding to each fixel. FC was derived from the warp fields computed during the registration of individual FODs to the template space. The FC values were then log-transformed for downstream analysis to ensure that the data were centered around zero. A combined measure incorporating both the above metrics, that is, FDC, was computed as the product of FD and FC (Dhollander et al., 2021). Finally, whole-brain probabilistic tractography was performed on the FOD template where initially 20 million streamlines were generated and subsequently filtered to 2 million streamlines using spherical deconvolution informed filtering of tractograms to reduce reconstruction bias (R. E. Smith et al., 2013). Connectivity-based fixel enhancement (CFE) using the template tractogram and nonparametric permutation testing with 5,000 permutations were performed for statistical analysis (Raffelt et al., 2015).

### DTI, FW-DTI, and DKI processing

**Figure 1.**
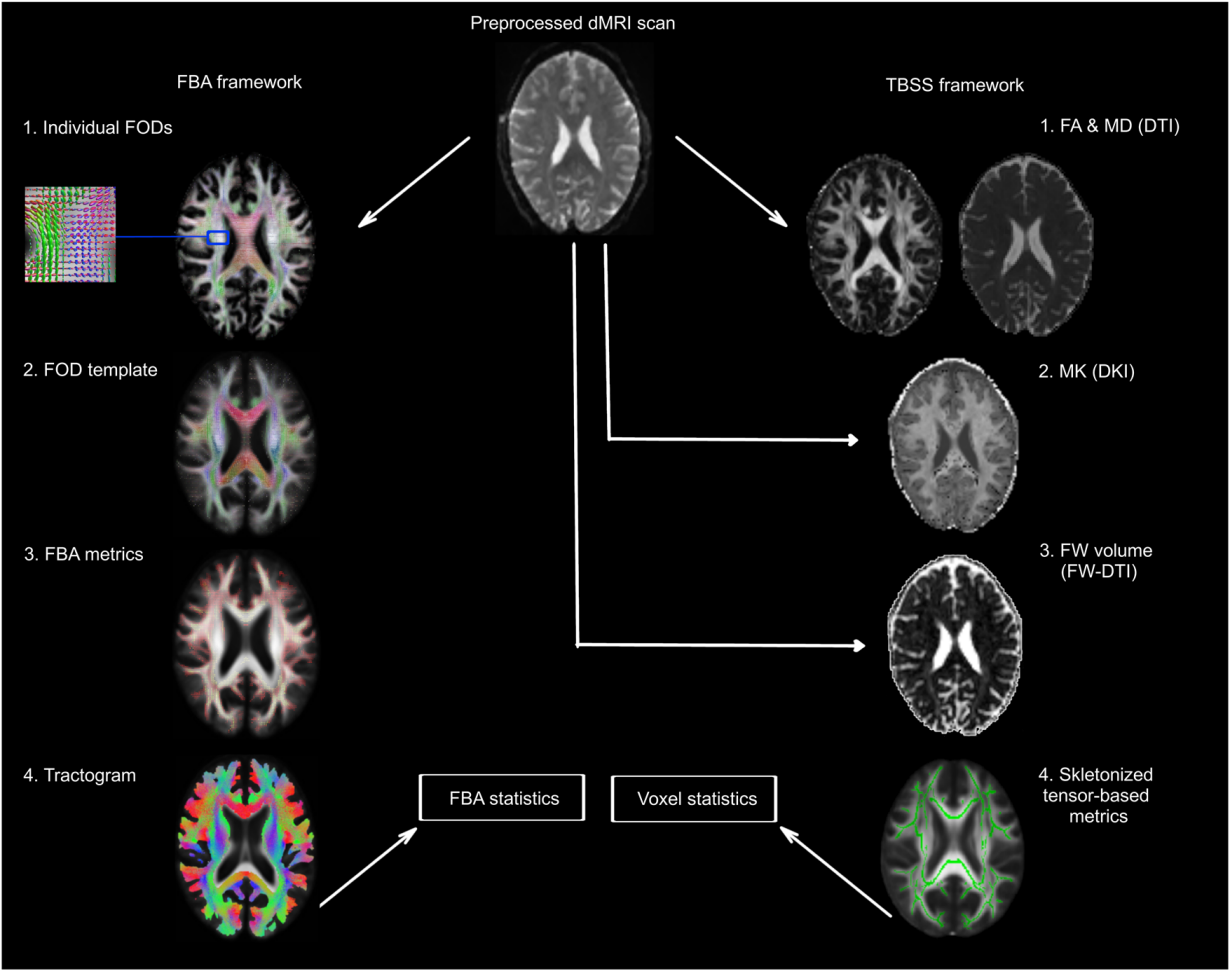
Overview of key processing steps of dMRI scans. Preprocessed dMRI data were used in FBA (left) and TBSS (right) frameworks. The preprocessing steps were identical for both approaches. Note that for DTI and FW-DTI, volumes with high *b* values were discarded from the preprocessed data. Individual FODs were obtained using MSMT-CSD and coregistered to a study-specific FOD template. Following the estimation of FBA metrics (FD, FC, and FDC) for each warped FOD image, a whole-brain tractogram was generated to conduct FBA statistics using CFE. Tensor-derived metrics including FA, MD, MK, and FW volume were obtained using DTI, DKI, and FW-DTI respectively. FA and MD images were also computed for DKI and FW-DTI but not displayed here. Voxel-wise analysis using TFCE was performed after the coregistration of DTI-derived metrics to the template space and their subsequent skeletonization.

To compare the results from FBA-derived metrics with more commonly used voxel-averaged measures, the preprocessed dMRI data were additionally fitted with tensor-derived models. Given that DTI and FW-DTI are traditionally applied to dMRI data with a lower number of gradient directions and *b* values, the 64 volumes with *b* = 2,500 s/mm^2^ were not included in these analyses. DTI-derived metrics including FA and MD were quantified via the “*dipy_fit_dti*” command of DiPY using weighted least squares regression. FW-DTI measures, that is, FA_t_, MD_t_, and FW images were estimated with a bitensor model that has been described previously (Pasternak et al., 2009), implemented using an in-house MATLAB script. Since DKI requires multishell data, all volumes of the preprocessed dMRI data were included in this analysis. DKI parameters such as FA(DKI), MD(DKI), and mean kurtosis (MK) were calculated using the DIPY module “*reconst.dki*” (Henriques et al., 2021). Afterward, all individual tensor-derived maps were projected onto a standard space and skeletonized using the tract-based spatial statistics (TBSS) toolbox in FSL (S. M. Smith et al., 2006). Voxel-wise analysis on the skeletonized tensor-derived metrics was conducted using FSL “*randomise*” function with threshold-free cluster enhancement (TFCE) and 5,000 permutations. An outline of the processing scheme for dMRI metrics is depicted in Figure 1.

### PET acquisition and processing

Aβ-PET images were acquired on a GE Discovery MI scanner (General Electric Medical Systems) 90–110 min after the injection of [18F] flutemetamol. Tau-PET scans using [18F] RO948 were obtained on the same scanner 70–90 min postinjection as previously described (Leuzy et al., 2020). PET and dMRI data acquisitions were on average < 3 months apart from each other (time gap between dMRI and Aβ-PET and dMRI and tau-PET: mean = 2.7; range, 0–23 months and mean = 2.5; range, 0–22 months, respectively). Standardized uptake value ratio (SUVR) images were calculated using the pons and inferior cerebellar GM as the reference regions for Aβ- and tau-PET, respectively (Leuzy et al., 2020; Pichet Binette et al., 2022). The acquired T1-weighted scans were used for PET-image coregistration and template normalization. Global Aβ burden was calculated using a neocortical composite region of interest (ROI) including prefrontal, parietal, and temporal lateral regions as well as the anterior/posterior cingulate and precuneus (Lundqvist et al., 2013; Landau et al., 2014). Tau uptake was quantified primarily in the entorhinal cortex as one of the most representative regions for early tau accumulation. Nonetheless, other medial temporal lobe (MTL) regions, that is, hippocampus, amygdala, and parahippocampus were also assessed for tau load. Note that Aβ- and tau-PET scans were not available in a small subsample of Aβ-negative CU (8 and 1), Aβ-positive CU (5 and 4), and Aβ-positive MCI participants (8 and 2) individuals, respectively.

### Statistical analysis

Between-group differences in dMRI-derived metrics were assessed using GLM whose parameters were estimated via CFE or TFCE (see FBA, DTI, FW-DTI, and DKI processing). To explore the correlations between both Aβ- and tau-PET uptake and dMRI-derived metrics, multiple univariate linear regression analyses were performed in the Aβ-positive individuals (Aβ-positive CU and Aβ-positive MCI). Similarly, the relationship between cognition and FBA-derived metrics was examined at the whole-brain fixel level and using ROI-based regression analyses performed in R (version 3.5.2). For the ROI-based analysis, each cognitive domain was modeled as an outcome measure and the FBA metrics as predictors. All models were controlled for the potential confounding effects of age, sex, ICV, and WMH quantified by total lesion volume. Years of education were included as an additional nuisance covariate when testing the correlations with cognitive performance. Moreover, the “mediation” package in R was employed to assess whether the link between entorhinal tau load and memory performance was mediated by GM atrophy of this region or by fiber-specific WM alterations. Statistical significance was set at FWE corrected threshold of *p*_FWE _< 0.05 for both FBA and DTI-derived analyses. Likewise, the statistical threshold for the ROI-based correlation analysis between FBA metrics and different cognitive functions was set to a Bonferroni-corrected *p*-value of 0.05.

## Results

### Group differences in FBA- and tensor-derived metrics

#### Reduced FBA metrics in MCI patients

**Figure 2.**
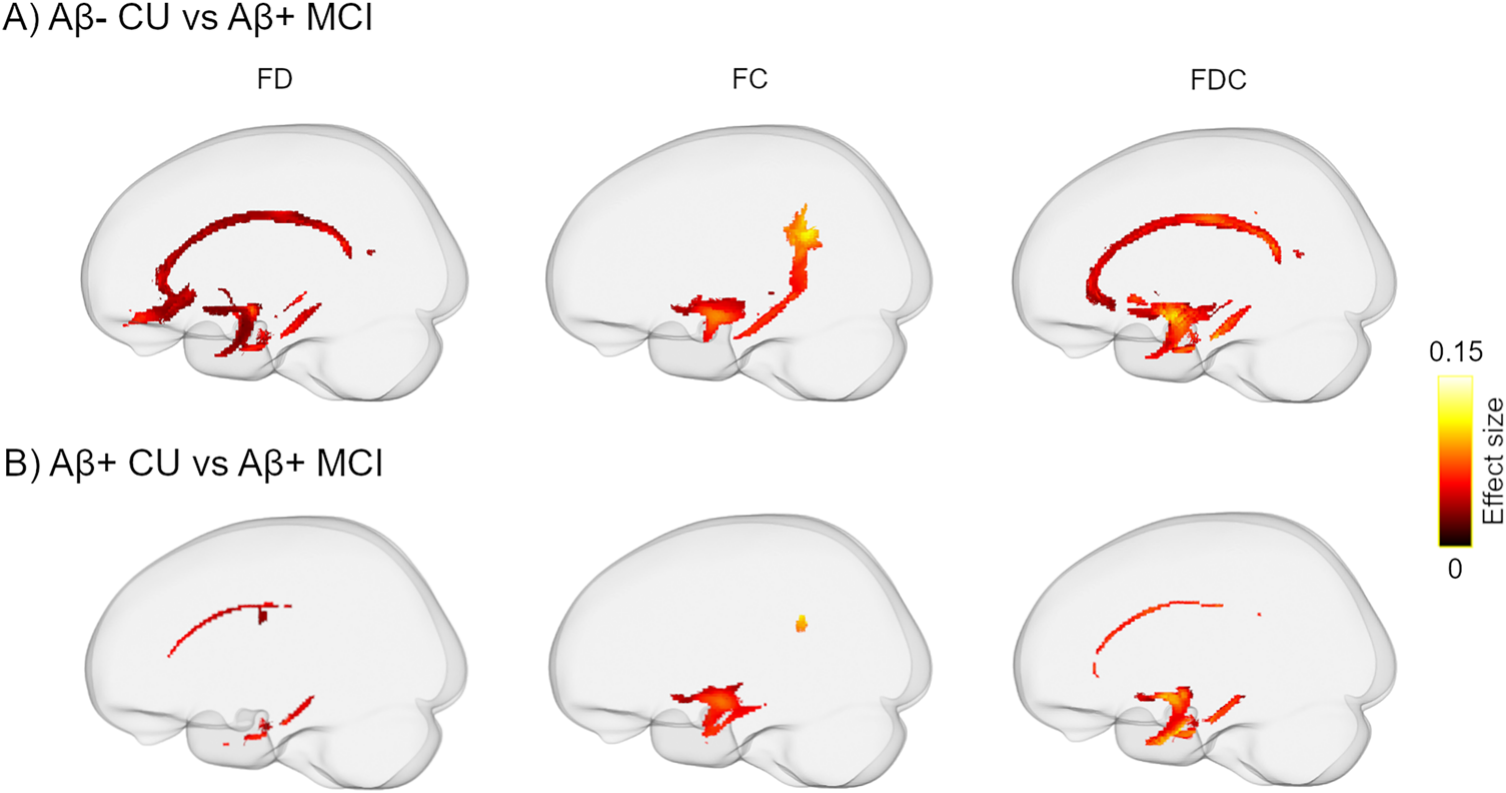
Comparison of FBA metrics between (***A***) Aβ-negative CU and (***B***) Aβ-positive CU individuals with Aβ-positive MCI patients. Streamlines were cropped from the template tractogram reflecting fixels with significantly reduced FD, FC, and FDC (left, middle, and right panels, respectively) in MCI patients. Significant streamlines are projected on the template glass brain (*p*_FWE _< 0.05).

Whole-brain FBA revealed a significant decrease across all three FBA metrics in Aβ-positive MCI patients compared with Aβ-negative CU individuals (Fig. 2*A*). Lower FD was found in the bilateral cingulum, parahippocampal (PH) parts of the cingulum bundle, uncinate fasciculi, anterior thalamic radiation, and forceps minor. FC exhibited a similar pattern of results although primarily restricted to the left hemisphere. Analogous findings were observed for FDC with a larger effect size. Likewise, when the Aβ-positive MCI group was compared with Aβ-positive CU participants a similar, although more restricted, pattern of reduction in FBA metrics was found (Fig. 2*B*). In contrast, no group differences in any of the FBA-derived measures were found between Aβ-negative and Aβ-positive CU individuals.

#### Differences in tensor-derived metrics in MCI patients

**Figure 3.**
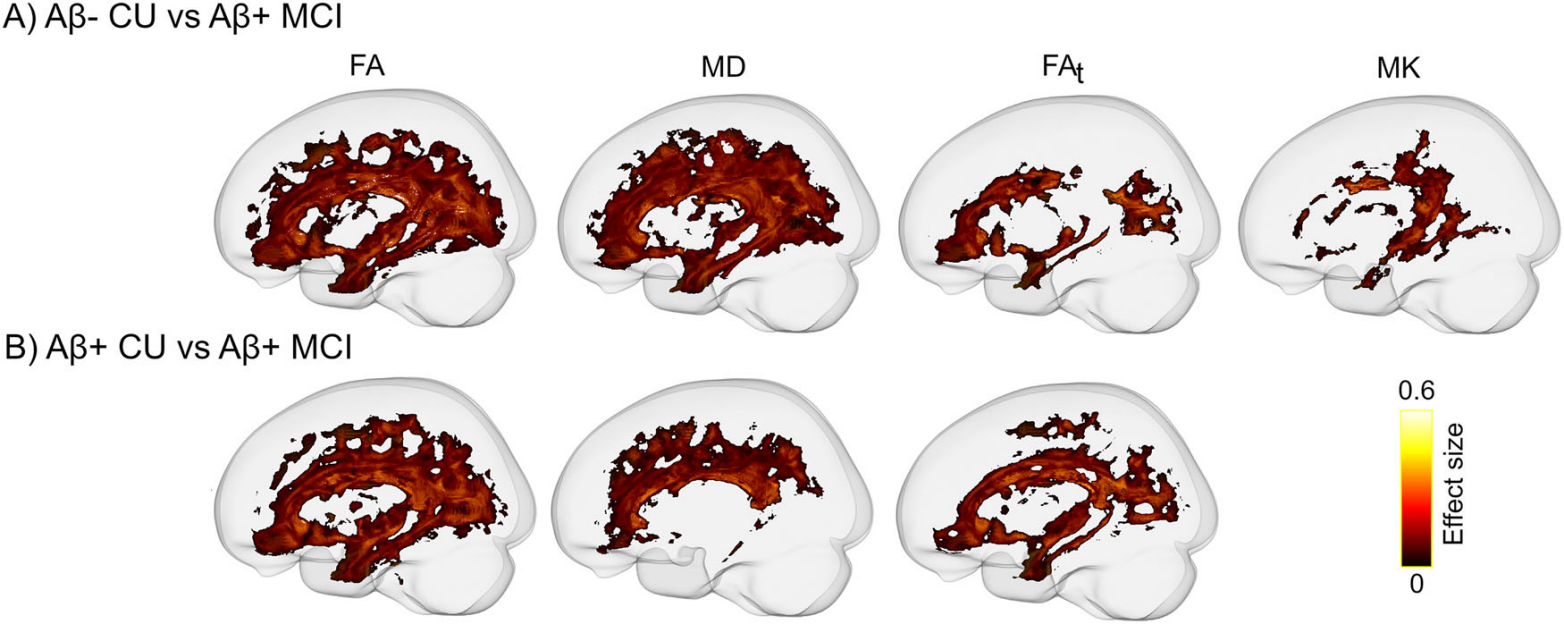
Voxel-wise comparison of tensor-derived metrics between (***A***) Aβ-negative CU and (***B***) Aβ-positive CU individuals with Aβ-positive MCI patients using TBSS. While FA, FA_t_, and MK are decreased, substantially increased MD is observed in MCI patients (*p*_FWE _< 0.05). WM tracts with significantly altered metrics are projected on the template glass brain.

Voxel-wise analysis using TBSS demonstrated a widespread decrease in FA and an extensive increase in MD when comparing Aβ-positive MCI patients with both Aβ-negative and Aβ-positive CU individuals (Fig. 3). Performing the same comparison using FW-DTI and DKI metrics revealed statistically significant differences only when employing FA_t_ and MK. Reduction in FA_t_ was found when comparing Aβ-positive MCI patients with both CU groups. However, MK reduction was observed only when the Aβ-positive MCI patients were compared with Aβ-negative CU (Fig. 3). No significant differences between Aβ-negative and Aβ-positive CU individuals were found in any of the tensor-derived metrics.

### Correlation between dMRI-derived metrics and AD molecular pathology

#### Higher MTL tau load is only associated with decreased FC in the parahippocampal segment of the cingulum bundle

**Figure 4.**
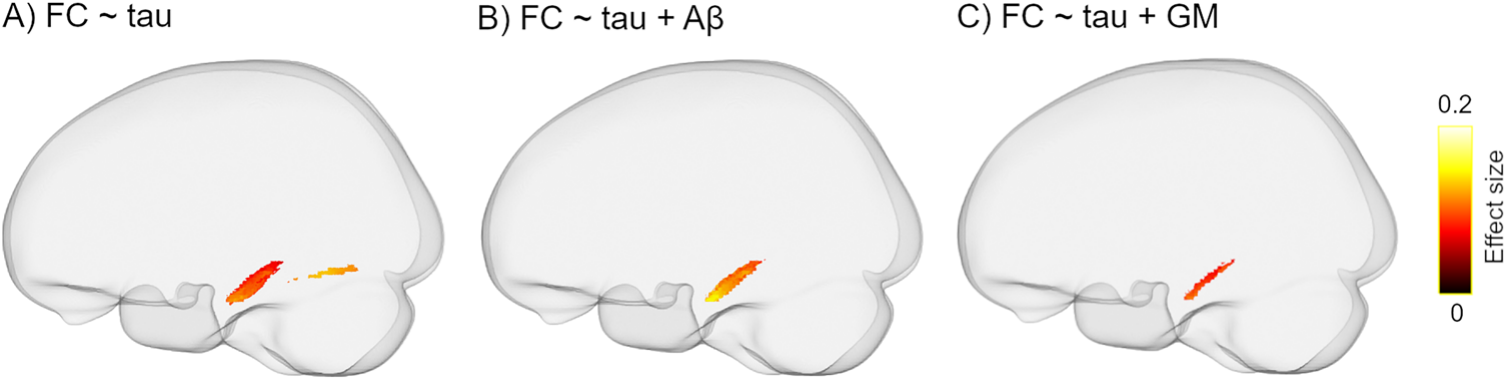
The inverse relationship between entorhinal tau uptake and FC. ***A***, Higher levels of entorhinal tau are accompanied by lower FC in the parahippocampal segment of the cingulum tract and inferior longitudinal fasciculus. The observed correlation remains significant although slightly decreased after controlling for (***B***) global Aβ load and (***C***) GM volume of the entorhinal cortex. Significant streamlines are displayed on the template glass brain (*p*_FWE _< 0.05). Note that the regression models were controlled for age, sex, WMHs, and ICV in ***A–C*** but are not shown in the figure legend for the sake of brevity.

**Figure 5.**
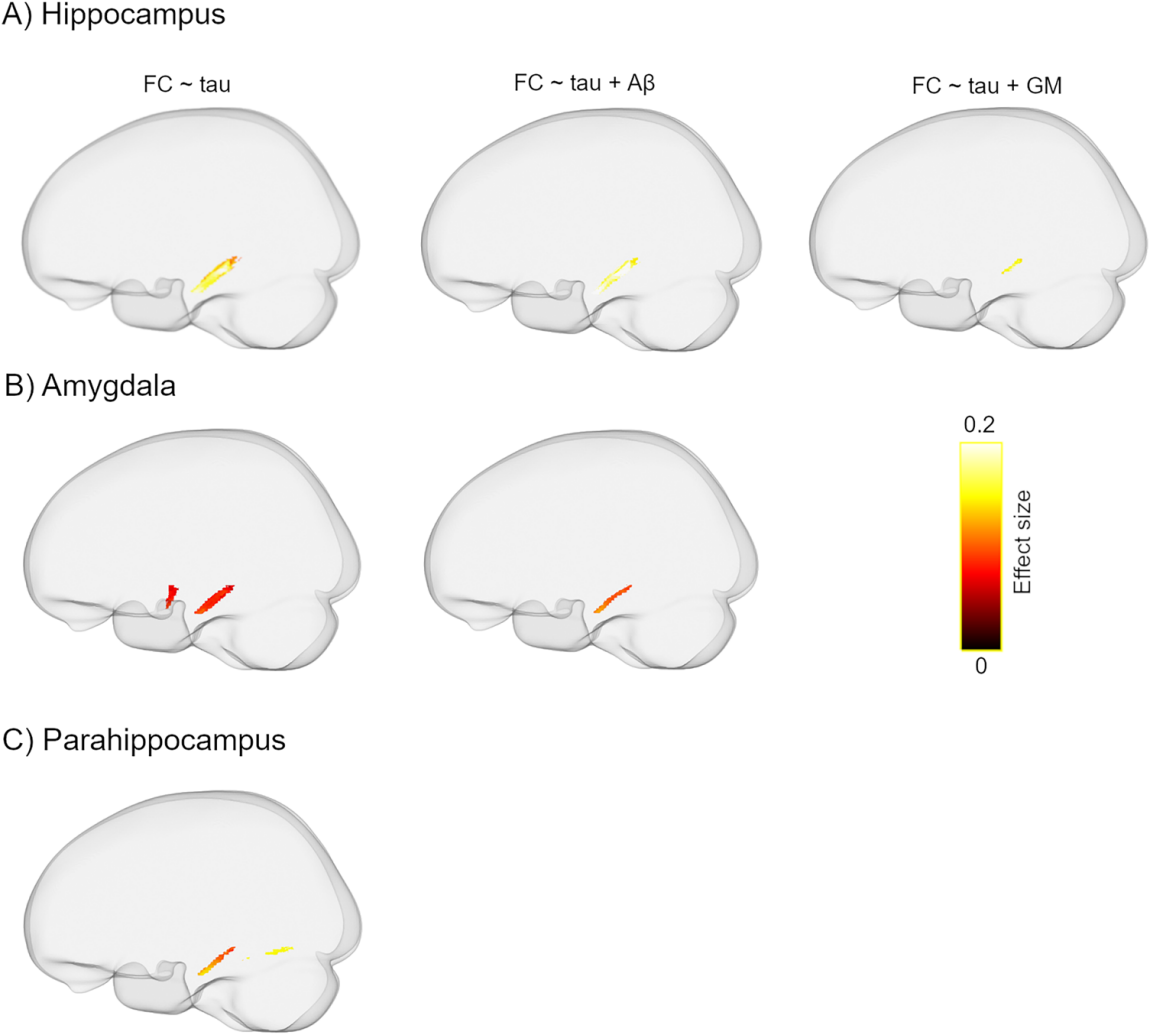
Correlation of FC and tau load in (***A***) hippocampus, (***B***) amygdala, and (***C***) parahippocampus. With increasing tau burden in medial temporal lobe regions, FC decreases primarily in the parahippocampal part of the cingulum (*p*_FWE _< 0.05). Note that after controlling for the global amyloid load (middle panel), the tau-FC correlation shrinks in the amygdala and becomes insignificant in the parahippocampal cortex. Similarly, the observed correlations markedly decrease in the hippocampus and disappear in the other two regions following additional correction for the corresponding GM volume (right panel). Note that all models are additionally adjusted for the confounding effects of age, sex, WMHs, and ICV.

Elevated tau load in the entorhinal cortex was associated with lower FC almost exclusively in the bilateral parahippocampal portions of the cingulum bundle (Fig. 4*A*). The observed findings remained largely intact following additional adjustment for global Aβ load (Fig. 4*B*). A further sensitivity analysis including the GM volume of the entorhinal cortex in the model showed consistent, although partially less widespread results (Fig. 4*C*). Similarly, increased tau-PET uptake in the other MTL regions was correlated with decreased FC mainly in the parahippocampal part of the cingulum (Fig. 5). Note that, except for FC, no significant correlation was found between other dMRI metrics and tau pathology. Aβ-PET uptake in the neocortical composite ROI was not correlated with any of the FBA- and DTI-derived measures.

### Correlations between tau-related WM alterations and cognitive performance

#### Decreased FBA metrics are correlated with memory decline

**Figure 6.**
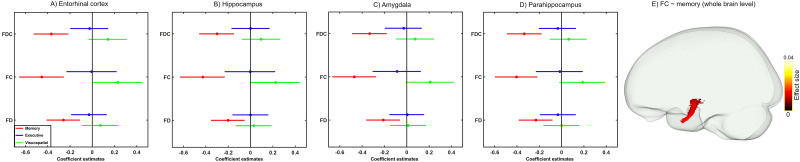
Associations of fiber-specific WM changes with cognition using ROI-based (***A–D***) and whole-brain FBA (***E***) analyses. Dot-whisker plots (***A–D***) show that all FBA metrics in WM masks that indicate a significant association with tau load in the MTL regions are correlated with memory deficits (Bonferroni-corrected *p* < 0.05). ***E***, At the whole-brain level, reduced FC in the temporal segment of the uncinate fasciculus is linked to worse memory performance (*p*_FWE _< 0.05). Note that in the ROI-based analysis, the cognitive functions are treated as outcomes whereas in the whole-brain fixel analyses they are predictor variables. All the regression models are corrected for the effects of age, sex, WMH, ICV, and education.

**Figure 7.**
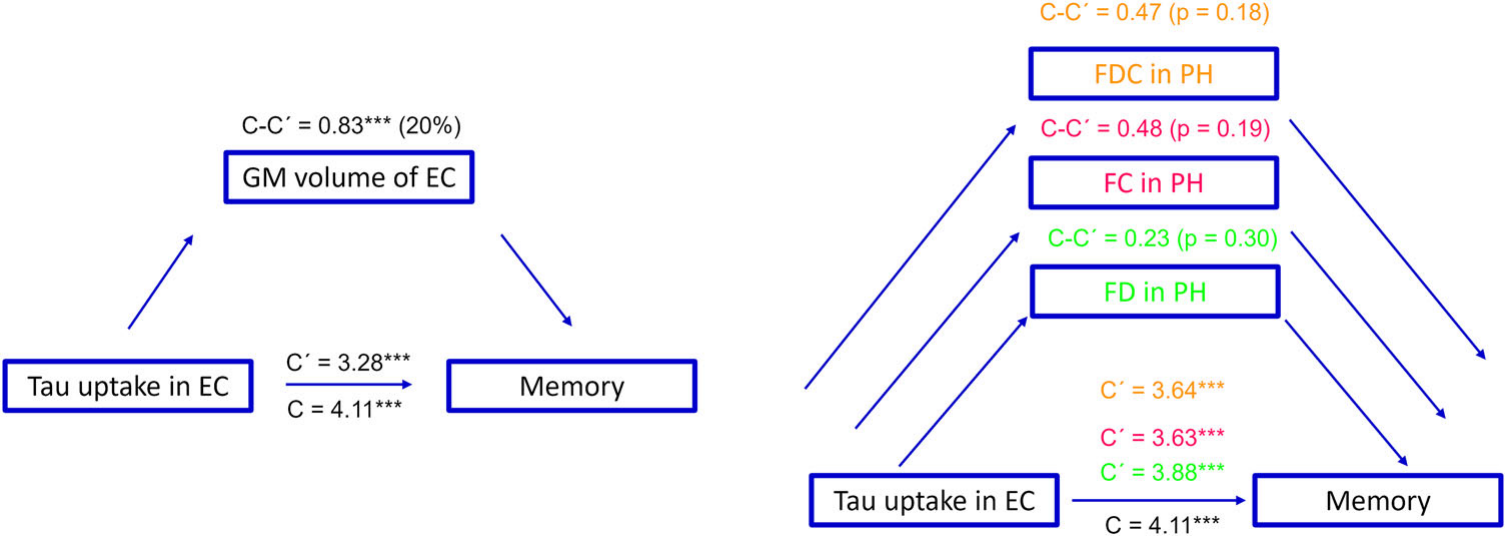
Flowchart illustration of the mediation analysis. The relationship between tau uptake in the entorhinal cortex (EC) is partially mediated by the GM volume of this region (mediation effect, 20%, left panel), but no mediatory effect is observed for the fiber-specific WM alterations in the parahippocampal (PH) segment of the cingulum (right panel). C presents the direct effect while C’ is the correlation strength after adjusting for the mediatory variables. C-C’ is, therefore, the mediation effect. The mediated effects of FBA metrics are condensed in a single schematic diagram, distinguished by distinct colors.

To assess the clinical relevance of tau-associated WM alterations and their impact on cognition, we obtained the mean values of each FBA metric for all Aβ-positive participants from a mask of fixels showing a significant correlation with the entorhinal tau load (Fig. 4*A*). Worse memory performance correlated with a reduction in all three FBA metrics (*β* = −0.25, −0.45, and −0.36 and Bonferroni-adjusted *p* = 0.002, 3.9 × 10^−5^, 1.9 × 10^−5^ for FD, FC, and FDC, respectively; Fig. 6*A*). No relationship was found between other cognitive domains and FBA metrics (Bonferroni-adjusted *p* > 0.05) although a positive correlation between FC and visuospatial performance was close to the significance threshold (*β* = 0.22, uncorrected *p* = 0.039, Bonferroni-adjusted *p* = 0.11; Fig. 6*A*). Similar findings were obtained when assessing the correlation between cognition and fiber-specific WM abnormalities related to tau accumulation in other MTL regions (Fig. 6*B–D*). To test whether the observed associations are influenced by the selected masks of significant fixels, we repeated the correlation analysis at the whole-brain level. In line with our ROI-based results, poor memory performance was correlated with reduced FC in the temporal segment of the uncinate fasciculus (Fig. 6*E*). Next, bootstrapped mediation analysis with 10,000 iterations was performed to investigate the possible effects of the GM volume of the entorhinal cortex and the observed tau-related WM alterations on the correlation between entorhinal tau accumulation and memory deficits. The GM volume of the entorhinal cortex partially mediated the tau-memory relationship (*β* = 0.83, CI = 0.1 to 1.34, *p* < 0.0001, 20% meditation effect). However, fiber-specific WM alterations did not have a significant mediation effect (*β* = 0.23 | 0.48 | 0.47, CI = −0.3 to 0.67 | −0.21 to 1.28 | −0.23 to 1.13, *p* > 0.05 for FD, FC, and FDC, respectively (Fig. 7).

Given that no tau-associated WM changes were observed using tensor-derived metrics (see above, Correlation between dMRI-derived metrics and AD molecular pathology), we did not further investigate the correlation of tensor-derived measures with cognitive performance as we were primarily interested in examining the clinical relevance of tau-related WM abnormalities on cognitive functions.

## Discussion

Combining advanced dMRI acquisition and analytical techniques with PET imaging and neuropsychological measurements, we sought to investigate the involvement of WM degeneration in the early stages of the AD pathological cascade. Using FBA, we observed degeneration at both micro- and macroscopic scales mainly localized in the cingulum, its parahippocampal segment, and frontal WM tracts in Aβ-positive MCI patients compared with Aβ-negative and Aβ-positive controls. Similar patterns were found using metrics derived from DTI, FW-DTI, and DKI with a larger spatial extent for the DTI approach. Further elevated tau-PET uptake in medial temporal lobe structures was specifically associated with macroscopic changes of the parahippocampal part of the cingulum as indicated by lower FC in Aβ-positive participants. The tau-related alterations in this WM bundle were correlated with a decline in memory performance. Importantly, this tau-WM correlation was not captured by the tensor-derived measures. This implies higher sensitivity of the FBA framework in detecting subtle WM degeneration related to tau accumulation, lending support to its utility as a potential biomarker for early detection and monitoring of tau-related AD progression.

Whole-brain FBA analysis demonstrated decreased FD, FC, and FDC in MCI patients in WM bundles associated with nodes of the default mode network connecting temporal, parietal, and frontal cortices. In the absence of resting-state fMRI data, these findings might provide indirect support for the network-based conceptualization of AD (Mito et al., 2018; Franzmeier et al., 2020; Giraldo et al., 2022). Remarkably, FD reduction was spatially more extensive than the decrease in FC suggesting that microstructural changes of the affected WM tracts might be more pronounced than the macroscopic alterations of the same structures. Alternatively, this may indicate that microscopic changes might precede macroscopic alterations. Additionally, our FBA findings partially overlapped with between-group differences obtained with other dMRI metrics. FA_t_ and MK showed a spatially comparable pattern of results, whereas FA and MD revealed widespread abnormalities in the MCI patients affecting many WM bundles. The limitations of DTI in the accurate estimation of the metrics in regions with crossing fibers or the contamination of these metrics by extracellular free water might result in inflated statistical results (Tournier et al., 2008; Pasternak et al., 2009). Nonetheless, the DTI-derived results of the current study are consistent with past evidence reporting WM alterations in early AD using DTI and its extensions (Teipel et al., 2010; Falangola et al., 2013; Gong et al., 2013; Metzler-Baddeley et al., 2014). By disentangling microstructural alterations from macroscopic WM changes, FBA provides fiber-specific insight, clarifying the present and previous DTI results that are likely driven by a combination of these differences. However, given the inherent differences between FBA- and tensor-based metrics, caution should be exerted when comparing the results of these approaches.

Contrary to our expectation, we did not observe a significant increase in FW volume between Aβ-positive MCI patients and either of the control groups whereas there are reports of higher FW across several WM bundles in the AD continuum (Ji et al., 2017; Mishra et al., 2020). This discrepancy might arise from multiple factors including differences in the analysis approaches. While some of those studies applied an ROI-based analysis, we performed a voxel-wise comparison in our study. The latter approach, taking into account the values of individual WM voxels across the brain, requires a more stringent correction for multiple comparisons compared with ROI-based approaches. Furthermore, it has been shown that WMHs impact FW alterations (Kamagata et al., 2022). As such, most of the studies that have found elevated FW volume in the AD spectrum have employed a normal-appearing WM mask in their analysis (Ji et al., 2017; Dumont et al., 2019). In contrast, we took into account the potential confounding effects of WMHs in the statistical models. Such differences could have also contributed to the contrasting results.

No group differences were found in FBA- or DTI-derived metrics between Aβ-negative and Aβ-positive controls. While there is no study with direct comparison of FBA measures in those groups, conflicting results have been reported on group differences in DTI-derived metrics. Specifically, one study has shown increased MD in presymptomatic familial AD (Li et al., 2015) possibly due to more aggressive degeneration in the genetic form of the disease. On the contrary, another study has found no significant differences in FA and MD between Aβ-positive and Aβ-negative CU individuals (Fischer et al., 2015). Our findings are in line with the latter indicating that amyloid burden per se does not induce WM degeneration. These results also suggest that in the absence of cognitive impairment, WM changes might not be present or are too subtle to be captured with current dMRI techniques.

Apart from the above between-group comparisons, all the remaining analyses were conducted in a pooled group of Aβ-positive individuals, that is, Aβ-positive CU and Aβ-positive MCI patients as we were interested in examining the link between dMRI metrics and amyloid, tau, and cognition in in the AD continuum irrespective of the cognitive status of the participants. To investigate the correlations with tau deposition, we focused on regions in which tau accumulates early on in the disease process, namely, the MTL. Interestingly, higher levels of tau uptake within the entorhinal cortex and other MTL structures were accompanied by reduced FC mainly in the parahippocampal segment of the cingulum bundle. A parsimonious explanation for this finding is that increased tau burden provokes axonal loss and WM atrophy that is captured by changes in FC. Notably, the parahippocampal portion of the cingulum connects key regions in the medial temporal and medial parietal lobes that are known to be affected during the early phase of AD pathogenesis (Zhou et al., 2008). The additional correction for global amyloid load did not alter the observed correlation, supporting an Aβ-independent relationship between tau accumulation and WM degeneration. However, further adjustments for the GM volume of the MTL regions decreased the effect size suggesting that the tau-FC correlation is not entirely independent from GM atrophy. Consistent with our findings, prior works have identified a link between increased tau-PET uptake and WM alterations in temporoparietal pathways (Jacobs et al., 2018; Strain et al., 2018; Binette et al., 2021; Luo et al., 2021).

Noticeably, the subtle correlation with tau pathology was not detected by any of the tensor-based metrics. This is particularly striking given that DTI measures, that is, FA and MD showed spatially extensive WM abnormalities in Aβ-positive MCI patients compared with either of the CU groups (Fig. 3), indicating that despite being highly sensitive, tensor-derived measures may lack specificity which in turn affects their clinical relevance. Nonetheless, the high sensitivity of tensor-based metrics could be still advantageous in specific settings when the prediction of future cognitive decline is required regardless of the underlying etiology. In contrast, when it comes to specificity to the AD pathology, FBA metrics or at least FC appear to be superior as evidenced by our results on the association of tau deposition with dMRI measures (Figs. 4, 5).

The correlation of cognitive performance with FBA metrics in the same WM tracts (Fig. 6*A–D*) further supports the clinical relevance of our results suggesting that the deterioration of memory functions is at least partially related to both micro- and macrostructural changes in WM. Moreover, mediation analysis was employed to investigate the relative contribution of GM and WM degeneration to memory impairment. Our results revealed that GM atrophy in the entorhinal cortex rather than WM alteration of the parahippocampal segment of the cingulum bundle mediated the relationship between elevated tau load in the entorhinal cortex and worse memory performance. These findings suggest that although memory deficits are linked to a synergetic degenerative process affecting both GM and WM, GM atrophy seems to bear a more direct impact on the relationship between tau accumulation and the deterioration of memory performance. It is worth noting that these results were driven from a mask of fixels being correlated with tau load in the entorhinal cortex and other MTL areas (Figs. 4, 5). The rationale behind this mask selection was to investigate the clinical relevance of tau-related early WM alterations. Given that tau accumulation starts in the MTL regions, our selected mask indicates early WM changes. It is noteworthy that whole-brain analysis on the association of FBA metrics with memory impairment revealed analogous findings (Fig. 6*E*) albeit with a lower effect size. This confirms that the observed correlation is independent of the mask choice and further suggests that WM damage in the MTL structures impacts memory decline.

Given that none of the DTI-derived metrics correlated with tau uptake in the MTL regions, we did not further investigate the associations of those measures with cognition. However, ample evidence indicates strong correlations between compromised WM integrity determined by alterations in FA, MD, FW, MK, and axial kurtosis with cognitive impairment in AD (Bosch et al., 2012, Gong et al., 2013, Ji et al., 2017, Zavaliangos-Petropulu et al., 2019).

The lack of a correlation between amyloid accumulation and WM degeneration, as suggested by our results, is in agreement with previous reports using both DTI (Strain et al., 2018) and FBA (Mito et al., 2018). In contrast, other studies have shown either a negative or a positive correlation between FBA or tensor-derived metrics with global Aβ-PET uptake in different stages of AD (Binette et al., 2021; Luo et al., 2021; Benitez et al., 2022; Dewenter et al., 2023). This discrepancy might be explained by differences in the selection of participants or analytical approach. Given the extensive differences in dMRI metrics observed between Aβ-positive MCI and the Aβ-negative CU groups (Figs. 2, 3), the absence of an association between Aβ pathology and dMRI metrics in the Aβ-participants may suggest that, after a certain threshold, the Aβ-induced damage to WM plateau while alteration in dMRI metrics might be more influenced by tau aggregation.

**Figure 8.**
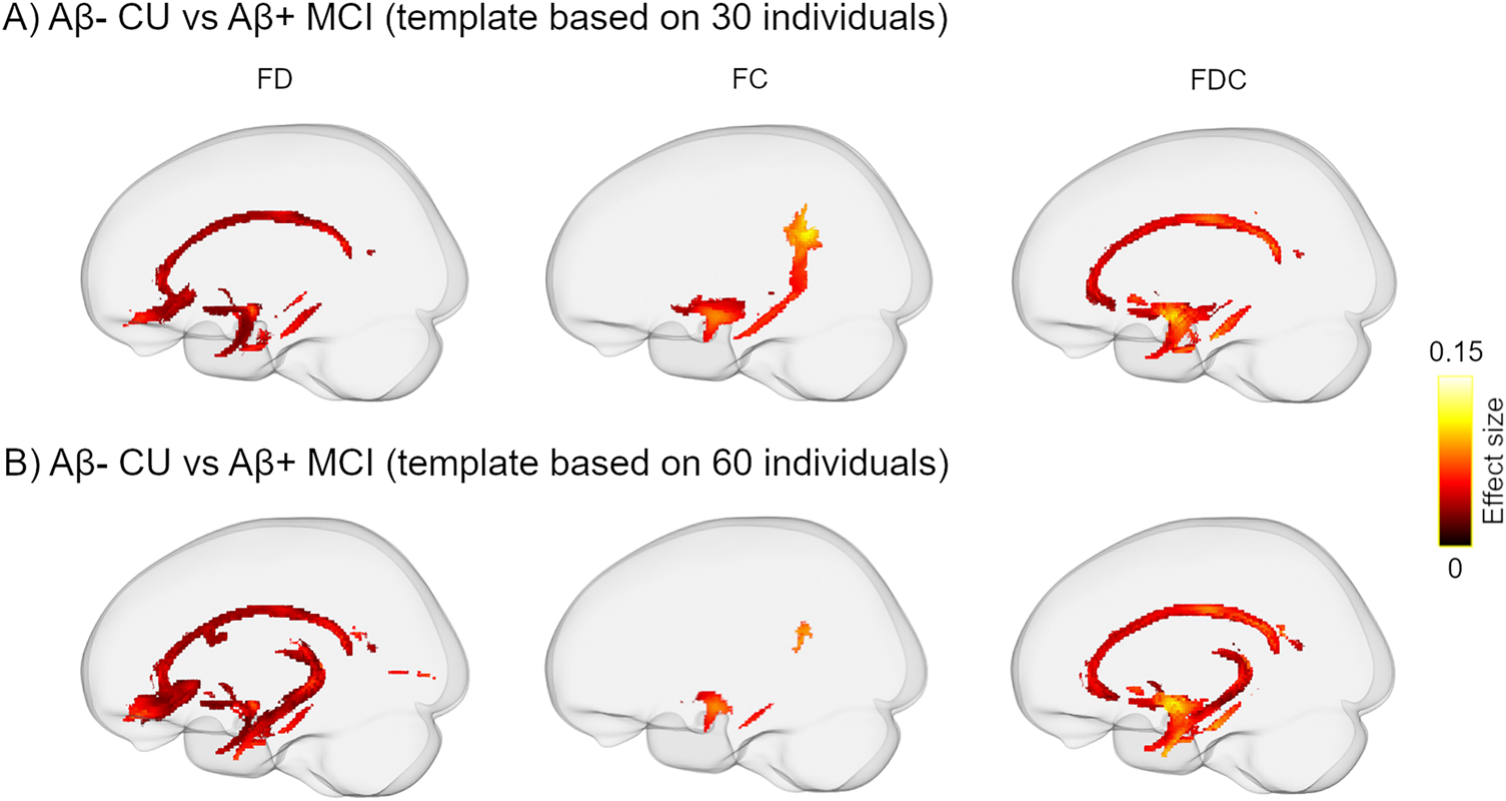
Comparison of FBA metrics between Aβ-negative CU and Aβ-positive MCI patients where the underlying FOD template was generated from (***A***) 30 or (***B***) 60 randomly selected individuals. The top panel (***A***) is identical to Figure 2*A*. The pattern of FBA reduction in the MCI group is largely similar in both panels indicating that the observed FBA alterations are not influenced by the number of selected FOD maps during the template generation.

It may be questioned why between-group differences in FBA and tensor-derived metrics (Figs. 2, 3) were not adjusted for additional covariates including global tau load and neuropsychological measurements. Tau levels correlate with the stage of the disease progression implying that Aβ-positive MCI patients inherently have higher global tau uptake than Aβ-positive and Aβ-negative CU individuals. Additionally, the MCI diagnosis in our cohort was based on patients’ performance on neuropsychological testing indicating that cognitive measures were implicitly embedded in the grouping variable. To avoid multicollinearity and interdependence between predictive variables, we opted not to incorporate them in the statistical models. Given that a critical step of whole-brain FBA analyses is the registration to a template space, the choice of the template itself could affect the results. We took an approach already employed by other FBA-based studies (Mito et al., 2018; Zarkali et al., 2020) and followed the recommended guidelines of MRtrix3. Nevertheless, to verify that the findings were not affected by the specific template of choice, we repeated the FBA processing by creating a new template using 60 participants (20 from each group) and compared FBA metrics between Aβ-negative CU and Aβ-positive MCI individuals. The pattern of results in all three FBA metrics (Fig. 8) was analogous to Figure 2*A* suggesting that FBA changes in the MCI group are largely independent of the number of selected FOD maps for template generation.

Since inaccuracies in the estimation of the response function can affect FOD characteristics and downstream FBA estimates, we acquired multishell dMRI with sufficient angular resolution and applied MSMT-CSD to improve the accuracy of the estimated response functions and subsequent FODs and FBA metrics (Dhollander et al., 2021). However, the angular deviations of the FOD peaks in multishell data may depend on the signal-to-noise ratio (SNR) level that is impacted by pathology (Guo et al., 2021). This might influence the interpretation of changes in FBA metrics. To ensure that the results are not biased by low SNR, we excluded 91 individuals with imaging artifacts and vascular and WM lesions (see Materials and Methods).

The cross-sectional nature of this study did not allow us to determine the temporal relationship between WM alterations, PET uptake, and cognitive measurements. Future studies are, thus, warranted to elucidate the longitudinal correlations between WM degeneration, molecular pathology, and clinical performance.

Leveraging a large sample size, multishell dMRI data acquisition with high angular resolution, and multiple diffusion models beyond DTI in conjunction with fixel-based and voxel-wise analyses, the present study provides an exhaustive evaluation of WM alterations and its relationship with the underlying pathology and core cognitive symptoms in the AD continuum. In conclusion, our results demonstrate that fiber-specific WM degeneration revealed by FBA is closely linked to tau accumulation and memory impairment even in the early stages of AD.

## Data Availability

Anonymized data will be shared by request from a qualified academic investigator for the sole purpose of replicating procedures and results presented in the article and as long as data transfer is in agreement with EU legislation on the general data protection regulation and decisions by the Ethical Review Board of Sweden and Region Skåne, which should be regulated in a material transfer agreement.
